# Commissioning and initial stereotactic ablative radiotherapy experience with Vero

**DOI:** 10.1120/jacmp.v15i2.4685

**Published:** 2014-03-06

**Authors:** Timothy D. Solberg, Paul M. Medin, Ezequiel Ramirez, Chuxiong Ding, Ryan D. Foster, John Yordy

**Affiliations:** ^1^ Department of Radiation Oncology University of Pennsylvania Philadelphia PA; ^2^ Department of Radiation Oncology University of Texas Southwestern Medical Center Dallas TX USA

**Keywords:** stereotactic ablative radiotherapy (SABR), stereotactic body radiation therapy (SBRT), commissioning, image‐guided radiotherapy

## Abstract

The purpose of this study is to describe the comprehensive commissioning process and initial clinical performance of the Vero linear accelerator, a new radiotherapy device recently installed at UT Southwestern Medical Center specifically developed for delivery of image‐guided stereotactic ablative radiotherapy (SABR). The Vero system utilizes a ring gantry to integrate a beam delivery platform with image guidance systems. The ring is capable of rotating $\pm \;60^{{}^{\circ}}$ about the vertical axis to facilitate noncoplanar beam arrangements ideal for SABR delivery. The beam delivery platform consists of a 6 MV C‐band linac with a 60 leaf MLC projecting a maximum field size of $15\times 15\;{\text{cm}}^{2}$ at isocenter. The Vero planning and delivery systems support a range of treatment techniques, including fixed beam conformal, dynamic conformal arcs, fixed gantry IMRT in either SMLC (step‐and‐shoot) or DMLC (dynamic) delivery, and hybrid arcs, which combines dynamic conformal arcs and fixed beam IMRT delivery. The accelerator and treatment head are mounted on a gimbal mechanism that allows the linac and MLC to pivot in two dimensions for tumor tracking. Two orthogonal kV imaging subsystems built into the ring facilitate both stereoscopic and volumetric (CBCT) image guidance. The system is also equipped with an always‐active electronic portal imaging device (EPID). We present our commissioning process and initial clinical experience focusing on SABR applications with the Vero, including: (1) beam data acquisition; (2) dosimetric commissioning of the treatment planning system, including evaluation of a Monte Carlo algorithm in a specially‐designed anthropomorphic thorax phantom; (3) validation using the Radiological Physics Center thorax, head and neck (IMRT), and spine credentialing phantoms; (4) end‐to‐end evaluation of IGRT localization accuracy; (5) ongoing system performance, including isocenter stability; and (6) clinical SABR applications.

PACS number: 87.53.Ly

## I. INTRODUCTION

Stereotactic radiosurgery (SRS) has been an effective modality for the treatment of benign and malignant cranial disease for over 60 years. Increasingly, in the stereotactic approach, ablative doses of radiation delivered in a highly focused manner to a target of interest, is being applied in a number of extracranial disease sites. Stereotactic ablative radiotherapy (SABR) holds significant potential for improving tumor control rates across a range of locations and histologies. Initial clinical results from prospective single institution, and more recently, multi‐institutional clinical trials of SABR have documented high rates of tumor control coupled with a low incidence of serious toxicity despite the high‐dose fractions of radiation being delivered. In the setting of early stage lung cancer, three‐year local control rates of 88% to 97% have been reported.^1^, ^2^, ^3^, ^4^, ^5^, ^6^, ^7^, ^8^, ^9^, ^10^, ^11^

SABR requires the use of technology at a standard above that routinely considered necessary for conformal radiotherapy and IMRT applications.^12^, ^13^, ^14^ Furthermore, the processes often involve a number of disparate, but interconnected, elements including: immobilization, motion management, image guidance, small field dosimetry, and dose calculation through complex heterogeneities. The selection, installation, integration, testing, and clinical application of technology are critical to ensuring safe and effective SRS/SBRT delivery.^13^, ^14^

In July 2011, a Vero linear accelerator (BrainLAB AG, Feldkirchen, Germany) was installed at the University of Texas Southwestern Medical Center specifically to support our stereotactic ablative radiotherapy (SABR) programs. Vero is a joint venture between BrainLAB AG and Mitsubishi Heavy Industries, LTD, (Tokyo, Japan); in Japan the Vero is known as the MHI TM‐2000. The Vero utilizes a ring gantry to integrate beam delivery and kV and MV image guidance systems on a mechanically rigid platform.^15^, ^16^ Electrons are accelerated using an inline side‐coupled standing wave C‐band linac, 38 cm in total length including the gun/injector, powered by a klystron and operating at 5.7 GHz,^(16)^ somewhat above that of conventional S‐band medical linear accelerators (~3 GHz), but less than that of X‐band linacs such as the CyberKnife (Accuray Inc., Sunnyvale, CA). An aluminum flattening filter provides a useful beam of up to $15\times 15\;{\text{cm}}^{2}$. The Vero has no electromagnetic beam steering, so any necessary adjustments to flatness and symmetry are made by manually moving the flattening filter. The nominal maximum dose rate is 500 MU/min.

The MLC, designed specifically for the Vero, consists of 30 opposing pairs of 11 cm thick tungsten‐alloy leaves projecting a width of 5 mm, for a maximum field size of $15\times 15\;{\text{cm}}^{2}$, at an isocenter located 100 cm from the X‐ray source.^(17)^ Leaves have rounded ends, and are capable of full over‐travel and interdigitation. The maximum leaf speed is 5 cm/second. The Vero has a fixed primary collimator positioned upstream of the MLC; there are no movable jaws. The linac and MLC are mounted together as a unit to a two‐axis gimbal mechanism that can pivot the entire assembly $\pm \;2.4^{{}^{\circ}}$ independently in the in‐plane and cross‐plane directions (corresponding to ± 4.2 cm at isocenter) at maximum speed of $9^{{}^{\circ}}/s$. Dynamic tracking capabilities received FDA approval in October, 2012; capabilities of the dynamic tracking system have been described elsewhere.^18^, ^19^, ^20^, ^21^

The source (gantry) rotates $\pm \;185^{{}^{\circ}}$ within the ring at a maximum speed of $7^{{}^{\circ}}/s$, while the ring itself rotates $\pm \;60^{{}^{\circ}}$ about a vertical axis at a maximum speed of $3^{{}^{\circ}}/s$. An advantage of the ring gantry design is rigid mechanical stability of both the gantry and ring rotations; in a prior analysis, the Vero demonstrated an isocenter radius of 0.12 and 0.02 mm for rotations of the gantry and ring, respectively.^(22)^ The Vero couch is capable of translations in three dimensions but, in contrast to conventional linac couches, it is not capable of rotation about a vertical axis. Rather, this degree of freedom is accomplished through the rotation of the ring. In addition, the Vero is equipped with a commercial robotic couch top (ExacTrac Robotic 6D Couch; BrainLAB AG) which corrects for the longitudinal and lateral rotations. Characteristics of the robotic couch top have been described previously.^23^, ^24^

The image guidance system consists of two X‐ray tubes (Shimadzu Corp., Kyoto, Japan) mounted in the ring $\pm \;45^{{}^{\circ}}$ on either side of the MV source and operating at potentials of 40‐150 kV. X‐rays are incident on two amorphous silicon detectors (PaxScan 4030A; Varian Medical Systems, Palo Alto, CA) at a source‐to‐detector distance (SDD) of 187.6 cm, projecting a maximum field size of $18\times 22{\;\text{cm}}^{2}$ at the isocenter. Image guidance can be performed in two ways: using the two kV systems together in a stereoscopic manner,^(23)^ or by obtaining projection images every 0.5° through a 225° rotation (CW from 320° to 175° or CCW from 40° to 185°) and reconstructing a volume (CBCT) using either source‐detector pair. The maximum CBCT field of view (FOV) is 20 cm in diameter by 15 cm long. The system can also be operated in stereoscopic fluoro mode for real‐time visualization and verification of dynamic tracking. The localization capabilities of both 2D/2D and CBCT systems were assessed as part of this work and are described below. The Vero system is also equipped with an amorphous silicon electronic portal imaging detector (EPID) (RID1640; Perkin Elmer, Santa Clara, CA) permanently mounted in the ring (i.e., not retractable) at an SDD of 221.2 cm. The EPID is operated in 1024×1024 mode, and acquires continuously at a frame rate of 2 Hz.

Because of the unique degree of freedom of the ring rotation, the primary beam is capable of impinging on a much greater wall area than a conventional linac. For an installation in an existing room, there is a high likelihood that the width of the primary barrier is insufficient. As compensation, the Vero ring contains a beamstopper that attenuates the primary beam by an additional factor of $10^{3}$. For our installation, no additional shielding was required.

In this work we describe the clinical commissioning of the Vero system for SABR applications, using key AAPM and ASTRO documents to guide the process.^13^, ^14^ We focus specifically on beam data acquisition and dosimetric commissioning of the treatment planning system and end‐to‐end evaluation of IGRT localization capabilities. Specially designed anthropomorphic head and thorax phantoms were used for hidden target testing and to benchmark a Monte Carlo algorithm for SABR applications in lung and spine. The Radiological Physics Center lung, head and neck (IMRT), and spine credentialing phantoms were used to independently verify system performance.

## II. MATERIALS AND METHODS

### A. Beam data

Beam data acquisition is a common task performed routinely by medical physicists; the general process has been described in detail in the report of AAPM Task Group 106.^(25)^ Acquisition of beam data for SRS and SABR can be particularly challenging, however, due to the small size of the fields employed and the partial volume effects associated with many common dosimeters. Further, small photon beams exhibit a loss of lateral electronic equilibrium on the central axis, producing output factors that falloff rapidly for fields below 10 mm in diameter.^26^, ^27^, ^28^, ^29^ Centering of the dosimeter in the beam is also challenging, and improper alignment of the beam and detector can introduce significant errors.

The planning system for Vero, iPlan versions 4.1 and 4.5 (BrainLAB AG), require a number of specific beam measurements, including percent depth dose, diagonal profiles, relative output factors, and MLC transmission. If dynamic IMRT delivery is to be used, an assessment of the radiation leaf gap due to the rounded leaf ends is also required. For dose calculation in homogeneous media, a pencil beam (PB) algorithm is used. For dose calculation in heterogeneous media, iPlan employs a Monte Carlo (MC) algorithm based on the XVMC dose developed by Fippel and Kawrakow.^30^, ^31^ Fragoso et al.^(32)^ performed extensive benchmarking of the algorithm against measurement, albeit for a different treatment unit/MLC. For commissioning the Monte Carlo algorithm, iPlan requires additional beam measurements, including central and off axis profiles and output factors in air and water. A complete list of beam data requirements is shown in Table 1.

Percent depth dose data were acquired at source‐to‐surface distances (SSD) of both 90 and 100 cm using a model 31014 pinpoint chamber (PTW, Freiburg, Germany) for field sizes $2\times 2\;{\text{cm}}^{2}$ and greater. The 31014 has an inner diameter of 2 mm, thus acquired ionization data were shifted proximally by 0.6 mm. Data were smoothed using a least square algorithm with a sliding window of 4 mm. A stereotactic diode (Edge, Sun Nuclear Corp., Melbourne, FL) was used to measure PDD, again at 90 and 100 cm SSD, for the $1\times 1\;{\text{cm}}^{2}$ field size. To improve the signal‐to‐noise characteristics, all diode data were acquired three times and subsequently averaged. All PDD data were resampled in 1 mm increments.

**Table 1:** Summary of required beam measurements

	*Algorithm*	
*Parameter*	*Pencil Beam*	*Monte Carlo*
Central Axis Profiles	Percent Depth Dose in water at 100 cm SSD for square fields 1×1 to $15\times 15\;{\text{cm}}^{2}$	Percent Depth Dose in water at 90 cm SSD for fields 1×1 to $15\times x15\;{\text{cm}}^{2}$
		CAX profiles in air for source‐to‐detector distances (SDD) of 85 to 115 cm for fields 1×1 to $15\times 15\;{\text{cm}}^{2}$
Off‐Axis Profiles	Diagonal Radial Profile in water at 100 cm SSD at depths from 0.5 to 350 cm, for a fully open $15\times 15\;{\text{cm}}^{2}$ field	Inline and crossline profiles in water for 90 and 100 cm SSD at depths of 1.5, 10, and 20 cm, for square and rectangular fields 1×1 to $15\times 15\;{\text{cm}}^{2}$
		Inline and crossline profiles in air for source‐to‐detector distances (SDD) of 85, 100, and 115 cm for square and rectangular fields 1×1 to $15\times 15\;{\text{cm}}^{2}$
Scatter Factors	Measurements in water at 100 cm SSD and a depth of 10 cm for square fields 1×1 to $15\times 15\;{\text{cm}}^{2}$ relative to a $10\times 10\;{\text{cm}}^{2}$ field	Measurements in water at 90 cm SSD and a depth of 10 cm for square and rectangular fields 1×1 to $15\times 15\;{\text{cm}}^{2}$ relative to a $10\times 10\;{\text{cm}}^{2}$ field
		Measurements in air for a source‐to‐detector distance (SDD) of 100 cm for square and rectangular fields 1×1 to $15\times 15\;{\text{cm}}^{2}$ relative to a $10\times 10\;{\text{cm}}^{2}$ field
MLC Leakage	Measurement in water at 100 cm SSD and a depth of 10 cm with MLC completely closed relative to an open $15\times 15\;{\text{cm}}^{2}$ field	
Absolute Dose	Gy/MU in water at 100 cm SSD, at a depth of 10 cm, for a $10\times 10\;{\text{cm}}^{2}$ field	Gy/MU in water at 90 cm SSD, at a depth of 10 cm, for a $10\times 10\;{\text{cm}}^{2}$ field

Off‐axis profiles were measured per manufacturer's requirements. Diagonal (radial) profiles for a fully open $15\times 15\;{\text{cm}}^{2}$ field were acquired at an SSD of 100 cm at seven depths in water, ranging from 0.5 to 350 cm, using the model 31014 pinpoint chamber with the central electrode oriented perpendicular to the beam axis. Pinpoint chamber profiles were obtained along both diagonals and subsequently averaged for input into the planning system. For comparison, measurement of diagonal profiles was repeated using the Edge diode. The Monte Carlo algorithm also requires measurement of transverse profiles in the direction parallel (left‐right=“inline”) and perpendicular (head‐foot=“crossline”) to the direction of MLC leaf travel. Transverse profiles were measured at three depths (1.5, 10.0, and 20.0 cm) at both 90 and 100 cm SSD, for square and rectangular fields ranging from 1×1 to $15\times 15\;{\text{cm}}^{2}$. The pinpoint chamber was oriented with the smallest dimension parallel to the scan direction. Standard profile characteristics, flatness, symmetry, measured full width at half maximum (FWHM), and penumbra (distance from 80% to 20%) were calculated and compared with characteristics from other stereotactic linacs.

Scatter factors, $S_{\text{cp}}$, for square and rectangular fields ranging from 1×1 to $15\times 15\;{\text{cm}}^{2}$ were measured in water at an SSD of 100 cm and depth of 10 cm using both the pinpoint chamber and stereotactic diode. Three readings were acquired for each field size and subsequently averaged. Data were normalized to a $10\times 10\;{\text{cm}}^{2}$ field size. The Edge diode is effectively independent of variation in energy spectrum that is manifest in changing field size^(33)^ and, therefore, the procedure of referencing diode measurements to the reference $10\times 10\;{\text{cm}}^{2}$ field through a smaller intermediate field (i.e., “daisy‐chaining”) was not performed.

Additional data were acquired for beam modeling for the Monte Carlo algorithm, notably, central axis profiles, off axis profiles, and output factors in air. In‐air CAX data were acquired for a source‐to‐detector distance ranging from 85 to 115 cm. In‐air, off‐axis data were acquired in both transverse directions at distances of 85, 100, and 115 cm from the source. All in‐air scans were performed using the pinpoint chamber equipped with a brass build‐up cap suitable for 6 MV photons.

MLC leaf transmission was evaluated separately for each leaf bank. In each case, the abutting leaf junction was positioned at the maximum range on the MLC, ± 7.5 cm from the central axis. At this location, the fixed primary collimator sufficiently shields the junction. Measurements were performed using extended dose range (EDR2) film (Carestream Health, Inc, Rochester, NY) placed perpendicular to the beam axis at a distance of 100 cm from the source, at a depth of 5 cm in Solid Water. 10,000 MU were delivered to each closed‐MLC field. A third film was irradiated under the same conditions using a $10\times 10\;{\text{cm}}^{2}$ open field and 100 MU. Films were scanned and analyzed using a commercial film dosimetry system (RIT113 V5.0; Radiological Imaging Technology, Colorado Springs, CO). Optical density was converted to dose using a sensitometric curve with a dose range from 0 to 6 Gy. The average transmission was determined from the ratio of the dose of the fully closed to that of the fully open field over 80% of the open field area. Additional transmission measurements were performed using a 0.3 cc ionization chamber (PTW 31013) located on the central axis and irradiated under identical conditions (i.e., 100 cm source‐to‐detector distance, 5 cm depth in Solid Water).

### B. Dosimetric commissioning

Following beam data acquisition, the treatment planning system was fully commissioned to ensure accurate calculation of dose and monitor units. This involved a systematic comparison of calculation and measurement, ranging from simple configurations such as a single beam, to sophisticated arrangements of beams encompassing all modes of delivery encountered in clinical practice. Prior to commissioning, the Vero machine was configured for the iPlan treatment planning system using the Beam Profile Editor version 7.1 utility (BrainLAB AG), and measured beam data were subsequently entered. The radial factors required by the planning system (a radial factor is the derivative of the half transverse profile) and the parameters for the Monte Carlo virtual energy fluence model (VEFM)^(34)^ (Table 2) were provided by the vendor based on user measured data.

A stack of Solid Water, 30 cm×30 cm×22 cm high was scanned and imported into the planning system. The stack is drilled for the pinpoint chamber at a depth of 11 cm and can hold film in a coronal orientation at several depths. Single anterior beams ranging in field size from 2×2 to $15\times 15\;{\text{cm}}^{2}$ were simulated in iPlan, and the dose calculated at discrete points along the central axis was compared with the measured beam data. Subsequently, a series of treatment plans were calculated and mapped onto the phantom. Delivery techniques included simple open and irregularly shaped conformal beam arrangements, dynamic conformal arcs, multiple isocenters, and IMRT. Point dose and film measurements were compared with TP calculations and spanned a range of doses up to approximately 20 Gy.

**Table 2:** Monte Carlo virtual energy fluence model parameters. The VEFM consists of a primary and scatter photon source modeled as two‐dimensional Gaussian shapes, and an electron contamination source

*Source Parameter*	*Value*
Primary Photon Source Weight	96.2598%
Primary Photon Source Distance	0 mm
Primary Photon Source Sigma	0.6 mm
Scatter Photon Source Weight	3.50369%
Scatter Photon Source Distance	145 mm
Scatter Photon Source Sigma	11.295 mm
Electron Source Weight	0.23649%
Electron Source Distance	145 mm
Electron Source Radius	24.4 mm
Electron Mean Energy	1.33 MeV

**Figure 1:**
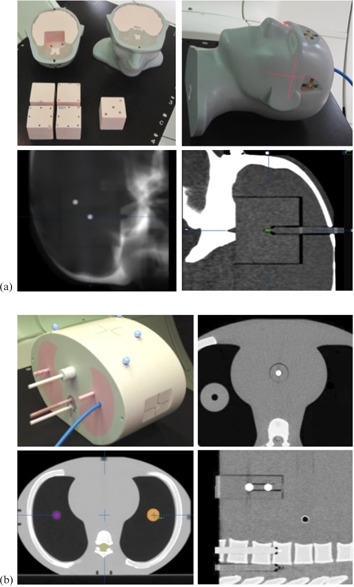
Head and neck end‐to‐end localization phantom (a), with various inserts for hidden targets, film and ion chamber dosimetry (top‐left), radiograph showing hidden targets (bottom‐left), and CT scan with pinpoint ionization chamber in place (bottom‐right). Thorax end‐to‐end localization phantom (b), showing the small and large unit density lung tumors into which dosimeters can be placed (bottom‐left) and hidden targets on axial and sagittal CT reconstructions (right). Holes to place ion chambers within the vertebral body and spinal cord are also visible in the sagittal reconstruction (bottom‐right).

At our institution, the Vero is used primarily for sophisticated treatments such as SABR. To assess accuracy and precision of both localization and dosimetric characteristics using image guidance in a clinically relevant setting, specialized anthropomorphic head and neck and thorax phantoms were designed and constructed (Integrated Medical Technologies, Troy, NY) (Figs. 1(a) and (b)). Each phantom contains radio‐opaque spheres for hidden‐target evaluation and multiple holes drilled to facilitate point dose measurements. In addition, the thorax phantom contains unit density targets imbedded in lung to allow direct verification of dosimetric calculations in heterogeneous media. Planning CT scans were obtained for both phantoms, and a series of treatment plans encompassing targets in the brain, lung, and spine were calculated and subsequently delivered to the phantoms. Point dose and planar film dosimetry were performed as described above.

Commissioning was further verified through the use of the Radiological Physics Center (RPC) thorax‐lung, spine, and head and neck IMRT phantoms. In each case, plans were developed based on the target coverage and OAR constraints required by the RPC; planning parameters and constraints are summarized in Table 3.

**Table 3:** Summary of target coverage and OAR constraints required for the RPC phantoms, with the planning/delivery technique in each case

*Phantom*	*RPC Thorax‐Lung*	*RPC Spine*	*RPC Head & Neck IMRT*
Planning / Delivery Technique	10 Noncoplanar Conformal Fields	13 Coplanar IMRT Fields	9 Coplanar IMRT Fields
Prescription / Target Coverage	>95% of PTV receives 6.00 Gy	>95% of PTV receives 6.00 Gy	>95% of 1° PTV receives 6.60 Gy, <1% of 1° PTV receives <6.14 Gy
	>99% of PTV receives 5.40 Gy	100% of PTV receives 5.25 Gy	>95% of 2° PTV receives 5.40 Gy, <1% of 2° PTV receives <5.02 Gy
	No hotpots outside PTV	No Dose >6.3 Gy outside PTV	
OAR Constraints	Maximum Spinal Cord Dose ≤5.00 Gy	<0.35 cc Spinal Cord receives >3.75 Gy	Maximum OAR Dose <4.5 Gy
	<37% Total Lung Volume receives >2.00 Gy	Maximum Spinal Cord Dose ≤5.25 Gy	Normal Tissue Maximum ≤7.26 Gy
	<33% Heart Volume receives >6.00 Gy	<1.2 cc Spinal Cord receives >2.63 Gy	
	<67% Heart Volume receives >4.50 Gy	<15 cc Heart receives >6.00 Gy	
	Maximum Heart Dose <4.50 Gy	Maximum Heart Dose <8.25 Gy	
		Esophagus Maximum Dose ≤6.00 Gy	
		<5 cc Esophagus receives >4.46 Gy	
		<1000 cc Total Lung receives >2.78 Gy	

### C. Localization accuracy

Assessment of localization accuracy in a manner that appropriately mimics the clinical SABR process is an essential aspect of device commissioning and ongoing QA. End‐to‐end target evaluation that incorporates CT, planning, and image guidance has become the standard for assessing overall system localization uncertainties.^12^, ^13^, ^14^, ^35^, ^36^, ^37^, ^38^, ^39^ To facilitate end‐to‐end evaluation of the Vero, the radio‐opaque spheres located within the head and thorax phantoms were identified on the respective planning CTs. An isocenter was positioned at the center of each sphere, and the resulting plan and reference datasets were transferred to the Vero through the R/V system. For each phantom, external marks were used to establish an initial position on the treatment couch; generally this initial position was within 2 cm of the desired isocenter in each direction. Based on anatomical data, the phantom was subsequently aligned to the machine isocenter using either stereo X‐ray (2D/2D) or CBCT (3D) image guidance. Automated image fusion was used to register the localization and reference images, followed by small manual adjustments. To guide 3D localization, contours from the planning CT are overlaid on the CBCT. With both techniques, the final alignment was determined by visual inspection of the images. Resulting translations and rotations were applied remotely to the Vero couch. The process was repeated a total of 28 times, 16 using CBCT image guidance and 12 using stereo x‐ray image guidance. For each trial, two or four beam's‐eye‐view EPID images were acquired corresponding to gantry angles of 0° (AP), 180° (PA), 90°, and/or 270° (lateral), using a field size of $2\times 2\;{\text{cm}}^{2}$. For each EPID image, a threshold was applied to isolate the open square field, from which the central pixel was subsequently identified. This process was repeated to isolate the hidden spherical target, and the two‐dimensional offsets between the field and target centers were recorded. This resulted in 28 independent measurements in the anterior‐posterior and left‐right directions, and 56 independent measurements in the superior‐inferior direction (since AP/PA and lateral images share a common sup–inf axis). All image analysis was performed using ImageJ.^(40)^

### D. Ongoing system performance

Since installation, numerous operational machine parameters have been tracked at regular intervals to assess ongoing system performance. Beam characteristics including output, flatness, and symmetry were assessed daily using a commercial QA device consisting of multiple ionization chambers (Daily QA3; Sun Nuclear Corporation). Coincidence of the lasers and kV and MV systems was assessed daily by the therapy staff, and repeated monthly as part of physics QA. Briefly, a $12\times 12\times 12\;{\text{cm}}^{3}$ phantom containing a radio‐opaque ball 1 cm in diameter is positioned on the treatment table and brought into coincidence with the Vero lasers. Stereoscopic X‐rays are obtained, the location of the ball relative to the kV imaging center is determined, and offset is subsequently corrected by translating the table in three dimensions. A series of 12 MV images at various gantry and ring angles are subsequently obtained, and the location of the ball relative to the MV imaging center is determined. All dosimetric and localization data were analyzed for compliance and for trends.

### E. Clinical applications

The majority of SABR treatments at our institution involve tumors of the lung, liver, and spine, though we have institutional protocols for breast, prostate, pancreas, and other disease sites. For tumors in the peripheral lung and liver, we employ a 3D conformal technique using multiple (≥10) noncoplanar beams; the rotating ring on the Vero provides opportunity for highly oblique beam directions. For tumors in the central lung and spine, fixed gantry IMRT is used; all spinal tumors are treated with 13 coplanar beams evenly distributed through 360°. For patients not enrolled in institutional or national study group trials, the most common prescriptions are as follows. Spine tumors are generally treated in a single fraction, with 20 Gy delivered to the GTV and 14 Gy to the CTV. Central and peripheral lung tumors are treated in 5 fractions of 10 Gy and 3 fractions of 18 Gy, respectively. Liver tumors are typically treated in 5 fractions of 10−12 Gy or on an institutional single fraction dose escalation protocol. The iPlan Monte Carlo algorithm is used for any tumors located in the thoracic region (lung and spine). Systematic use of normal tissue dose constraints is followed in all SABR treatments.^(41)^

## III. RESULTS

### A. Beam data

**Figure 2:**
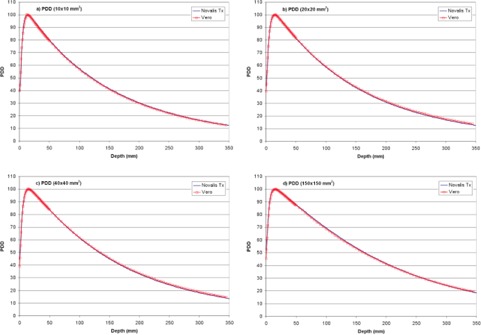
Vero percent depth dose for 1×1 to $15\times 15\;m^{2}$ fields, compared with similar data from a Novalis Tx unit.

Figure 2 shows percent depth dose for several field sizes, plotted against PDD data from another dedicated stereotactic linac (Novalis Tx; Varian Medical Systems and BrainLAB AG). The Vero PDD are similar to that of another commercial unit routinely used for SABR, specifically the Novalis Tx, which has a PDD of 85.9% at 5 cm depth for a $10\times 10\;{\text{cm}}^{2}$ field relative to 86.2% for the Vero.^(35)^ The Vero reference PDD (100 cm SSD, $10\times 10\;{\text{cm}}^{2}$ field size, 10 cm depth) is 66.7%.

**Figure 3:**
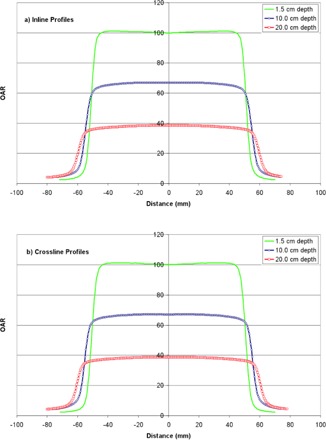
Inline profiles (a) and crossline profiles (b) at three depths in water, normalized to $D_{\text{max}}$.

Orthogonal profiles for a $10\times 10\;{\text{cm}}^{2}$ field measured at depths of 1.5, 10.0, and 20.0 cm are shown in Fig. 3. Data are normalized to 100% on the central axis at a depth of 1.5 cm. Profile characteristics, FWHM, flatness, symmetry, and penumbra are summarized in Table 4; data from an earlier characterization of the Vero are also included.^(17)^ As expected, penumbra in the crossline (left‐right) direction is slightly larger, due to the rounded leaf ends. Flatness and symmetry are within acceptable ranges, and penumbra is consistent with that of other stereotactic linacs. Glide‐Hurst et al.^(42)^ have reported penumbra values of 7.67 and 6.82 mm in the inline and crossline directions, respectively, for the Millennium 120 leaf MLC (5 mm leaf width) on a TrueBeam linac. In that work, profile measurements were performed using a 0.13 cc chamber, which the authors previously reported resulted in a blurring of the penumbra due to the relatively large diameter of the sensitive volume.^(35)^ Yin et al.^(43)^ reported penumbra values ranging from 2 mm to 3.5 mm for field sizes of 6 mm to 100 mm for the 52 leaf Novalis MLC; measurements were performed at a depth of 7.5 cm and an SSD of 92.5 cm. The authors subsequently reported a penumbra of 5.3 mm for a $10\times 10\;{\text{cm}}^{2}$ field for the HD‐120 MLC on a Novalis TX linac.^(44)^

**Figure 4:**
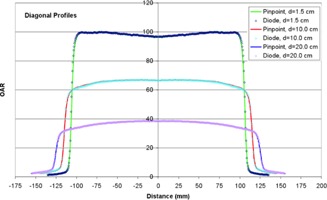
Diagonal profiles for a $15\times 15\;{\text{cm}}^{2}$ field at three depths in water, measured using a stereotactic diode and pinpoint chamber.

**Figure 5:**
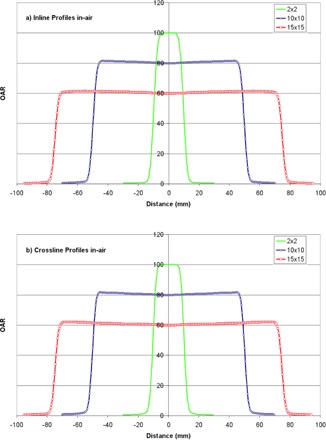
Inline profiles in air (a) for 2×2 to $15\times 15\;{\text{cm}}^{2}$ fields, normalized to the central axis (2×2), 80% of the central axis (10×10), and 60% of the central axis (15×15). Crossline profiles in air (b) for the same conditions.

Diagonal profiles for a $15\times 15\;{\text{cm}}^{2}$ field measured for the Vero at depths of 1.5, 10.0, and 20.0 cm are shown in Fig. 4, with pinpoint ionization chamber and diode data superimposed for comparison. Finally, in‐air profiles are shown in Figs. 5(a) and (b) for field sizes ranging from 2×2 to $15\times 15\;{\text{cm}}^{2}$; data for the 10×10 and $15\times 15\;{\text{cm}}^{2}$ fields are normalized to 80% and 60% of the maximum dose, respectively, to simplify visual analysis.

**Table 4:** Summary of beam profile characteristics from the current study, compared with those of a previous work (Nakamura et al.^(17)^)

		*Field Size (mm)*		*Flatness (%)*		*Symmetry (%)*			*Penumbra (mm)*		
Nominal Field Size (mm)	SAD (cm)	LR	HF	LR	HF	LR	HF	L	R	H	F
100.0	90	100.1	100.9	2.0	2.3	0.8	1.3	5.2	5.1	4.8	5.2
110.0	100	110.1	111.0	2.0	2.0	0.7	0.6	5.4	5.4	5.0	5.3
This work											
100.0	90	100.4	100.1	2.2	2.7	0.1	0.1	4.6	4.4	4.5	4.4

Nakamura et al.^(17)^

Scatter factors obtained using both a pinpoint chamber and diode are listed in Table 5. There is good agreement between the two detectors at all field sizes, except $1\times 1\;{\text{cm}}^{2}$, where partial irradiation of the chamber volume produces an artificially low reading.

**Figure 6:**
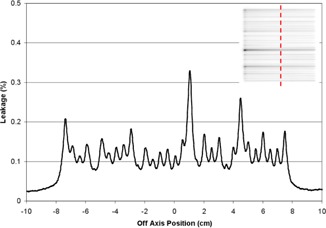
Leakage profile through the center of the closed MLC, as indicated.

Figure 6 shows the MLC leakage profile normalized to the open field central axis; interleaf leakage varies from approximately 0.08 to 0.35 percent. The average leakage over the entire field is 0.13 percent, which is similar to a value of 0.11 percent reported previously.^(17)^

**Table 5:** Total scatter factors, $S_{\text{cp}}$, as a function of square field size, measured using a diode and a pinpoint ionization chamber

*Nominal Field Size (mm)*	$S_{\text{cp}}$ *(pinpoint)*	$S_{\text{cp}}$ *(diode)*	*% Difference (pinpoint‐diode)*
10.0	0.704	0.745	−5.851
20.0	0.831	0.828	0.382
30.0	0.871	0.863	0.900
40.0	0.900	0.893	0.814
60.0	0.942	0.937	0.574
80.0	0.974	0.970	0.386
100.0	1.000	1.000	–
120.0	1.021	1.024	−0.314
150.0	1.043	1.053	−0.987

### B. Dosimetric commissioning

Table 6 shows the comparison of measured and calculated depth dose for square fields from 2×2 to $10\times 10\;{\text{cm}}^{2}$. Agreement is within 1% for all field sizes and all depths. A summary of commissioning plans comparing calculation with point dose measurements in Solid Water is presented in Table 7. The average difference is 0.16% across a wide range of field sizes, irradiation techniques, and prescribed/delivered doses. The maximum difference of 4.17% was observed in a spine SBRT case, planned and delivered using IMRT. In the Solid Water phantom, PB and MC algorithms agreed within 0.5%.

The anthropomorphic lung phantom contains two unit density targets, a “small” target 18 mm in diameter, and a “large” target 38 mm in diameter (Fig. 1(b), lower left). Each target is completely surrounded by lung material with an average relative electron density of 0.24. A summary of commissioning plans and point‐dose measurements for both targets, utilizing a variety of prescribed doses and irradiation techniques, is shown in Table 8. Calculations were performed using both the PB and MC algorithms. Overall, the MC algorithm demonstrates much better agreement with measurement than the PB algorithm, −0.11% versus −4.62% on average, respectively. The PB algorithm consistently overestimates dose by up to 12%, with the greatest disagreement observed for the small target (and small field sizes).

**Figure 7:**
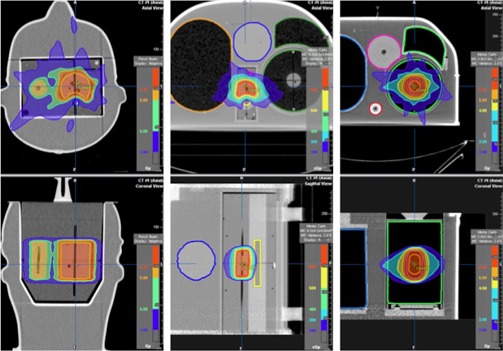
Planned dose distributions for RPC head and neck IMRT, spine and thorax phantoms (left to right).

Figure 7 shows treatment plans delivered as part of RPC thorax‐lung, spine, and head and neck IMRT phantom credentialing. Each of the phantom irradiations passed the credentialing process on the first attempt; results are summarized in Table 9.

**Table 6:** Comparison of measured and calculated percent depth‐dose data

*Field Size (cm^2^)*	*Depth (cm)*	*Measured (cGy)*	*Calculated (cGy)*	*% Diff*	*Field Size (cm^2^)*	*Depth (cm)*	*Measured (cGy)*	*Calculated (cGy)*	*% Diff*
2×2	2.0	98.32	98.00	0.33	4×4	2.0	98.79	98.00	0.80
	3.0	92.73	93.00	−0.29		3.0	93.97	94.00	−0.03
	4.0	86.96	87.00	−0.05		4.0	88.80	88.00	0.90
	5.0	81.74	82.00	−0.32		5.0	83.84	84.00	−0.19
	7.5	69.32	69.00	0.46		7.5	72.03	72.00	0.04
	10.0	59.08	59.00	0.14		10.0	61.81	62.00	−0.31
	12.5	50.36	50.00	0.71		12.5	52.99	53.00	−0.02
	15.0	43.09	43.00	0.21		15.0	45.46	45.00	1.01
6×6	2.0	99.00	99.00	0.00	10×10	2.0	98.94	99.00	−0.06
	3.0	94.21	95.00	−0.84		3.0	94.93	95.00	−0.07
	4.0	89.97	90.00	−0.03		4.0	90.55	90.00	0.61
	5.0	85.39	86.00	−0.71		5.0	86.20	86.00	0.23
	7.5	74.31	75.00	−0.93		7.5	76.15	76.00	0.20
	10.0	64.17	64.00	0.26		10.0	66.69	66.00	1.03
	12.5	55.32	56.00	−1.23		12.5	58.26	58.00	0.45
	15.0	47.72	48.00	−0.59		15.0	50.83	51.00	−0.33

**Table 7:** Summary of Solid Water commissioning plan calculations and point‐dose measurements

*Configuration*	*Planned Dose (Gy)*	*Measured Dose*	*% Difference*
4 field box $14\times 14\;{\text{cm}}^{2}$ square fields	2.504	2.502	−0.08
4 field box $10\times 10\;{\text{cm}}^{2}$ square fields	2.998	2.998	0.00
4 field box $8\times 8\;{\text{cm}}^{2}$ square fields	2.494	2.490	−0.16
4 field box $5\times 5\;{\text{cm}}^{2}$ square fields	2.506	2.470	−1.44
4 field box $4\times 4\;{\text{cm}}^{2}$ square fields	2.500	2.504	0.16
4 field box $2\times 2\;{\text{cm}}^{2}$ square fields	2.496	2.499	0.12
parallel‐opposed irregular fields	2.463	2.418	−1.83
5 field conformal irregular fields	4.050	3.978	−1.78
2 dynamic conformal arcs	2.510	2.481	−1.16
4 dynamic conformal arcs	4.028	4.037	0.22
3 dynamic conformal arcs	4.038	4.062	0.59
2 isocenters with dynamic conformal arcs	3.070	3.071	0.03
7 field IMRT mapped from patient plan	3.080	3.075	−0.16
5 field IMRT mapped from patient plan	6.480	6.545	1.00
10 field lung SBRT mapped from patient plan	13.850	13.980	0.94
13 field prostate IMRT mapped from patient plan	9.980	10.059	0.79
13 field prostate IMRT mapped from patient plan	5.000	5.011	0.22
12 field spine IMRT mapped from patient plan	20.620	21.479	4.17
12 field spine IMRT mapped from patient plan	4.210	4.262	1.24
13 field spine IMRT mapped from patient plan	11.627	11.660	0.28
Average (Standard Deviation) Difference			0.16 (1.32)

It should be emphasized that both the anthropomorphic lung and RPC phantoms were localized using image guidance. In this manner, dosimetric evaluation is performed in an end‐to‐end manner that precisely mimics the clinical treatment process.

**Table 8:** Summary of commissioning plans and point‐dose measurements in an anthropomorphic lung phantom

		*Planned Dose (Gy)*		*% Difference*	
*Configuration*	*Measured Dose (Gy)*	*PB*	*MC*	*PB*	*MC*
7 coplanar conformal beams – small lung target	3.03	3.47	3.12	−12.16	−3.13
11 coplanar conformal beams – small lung target	9.42	9.97	9.33	−5.57	0.94
11 noncoplanar conformal beams – small lung target	9.48	10.08	9.33	−6.01	1.58
6 coplanar conformal beams – large lung target	4.17	4.22	4.11	−1.23	1.56
7 coplanar conformal beams – large lung target	4.89	4.98	4.83	−1.73	1.26
11 noncoplanar conformal beams – large lung target	19.53	20.58	19.93	−5.01	−2.01
11 noncoplanar conformal beams – large lung target	18.61	19.53	18.97	−4.71	−1.90
11 noncoplanar conformal beams – large lung target	15.92	16.68	15.97	−4.56	−0.31
13 coplanar IMRT beams – T spine target	9.15	9.20	9.06	−0.59	1.02
				−4.62	0.11
Average (Standard Deviation) Difference				(3.46)	(1.80)

**Table 9:** Summary of RPC phantom irradiation results for the lung, IMRT head and neck and spine phantoms

*Phantom*	*Location*	*RPC vs. Institution*	*Criteria*
Lung Phantom	PTVTLDsup	0.96	0.92‐1.02
	PTVTLDinf	0.95	0.92‐1.02
	*Film Plane*	*Gamma Index*	*Criteria*
	Axial	97%	≥80%
	Coronal	96%	≥80%
	Sagittal	96%	≥80%
	Average over 3 planes	96%	≥85%
IMRT Head and Neck Phantom	Primary PTV sup. ant.	0.96	0.93−1.07
	Primary PTV inf. ant.	0.97	0.93−1.07
	Primary PTV sup. post.	0.99	0.93−1.07
	Primary PTV inf. post.	0.98	0.93−1.07
	Secondary PTV sup.	0.96	0.93−1.07
	Primary PTV inf.	0.96	0.93−1.07
	*Film Plane*	*Gamma Index*	*Criteria*
	Axial	100%	≥85%
	Sagittal	99%	≥85%
Spine Phantom	PTVTLDsupant	0.99	0.93−1.07
	PTVTLDinfant	1.01	0.93−1.07
	PTVTLDsuppost	1.01	0.93−1.07
	PTVTLDinfpost	1.02	≥85%
	*Film Plane*	*Gamma Index*	*Criteria*
	Axial	96%	≥85%
	Sagittal	100%	≥85%

### C. Localization accuracy

Results of the 28 independent hidden‐target tests are summarized in Table 10. Overall localization accuracy, characterized by one standard deviation, is 0.34 mm, 0.27 mm, and 0.27 mm in anterior‐posterior, lateral, and superior‐inferior directions, respectively. Maximum target errors are less than 1 mm in every direction. No significant differences were observed between CBCT and stereo X‐ray image guidance.

**Table 10:** Results from 28 independent end‐to‐end hidden0target tests, 12 performed using 2D/2D (ExacTrac) localization and 16 performed using cone‐beam CT localization

	*Hidden Target Offset (mm)*											
	*Anterior—Posterior*				*Right‐Left*				*Superior—Inferior*			
	*Ave*	*Min*	*Max*	*STD*	*Ave*	*Min*	*Max*	*STD*	*Ave*	*Min*	*Max*	*STD*
All Data (n=28)	0.00	−0.67	0.79	0.34	−0.26	−0.75	0.46	0.27	0.19	−0.34	0.73	0.27
2D/2D (n=12)	0.14	−0.67	0.79	0.37	−0.24	−0.60	0.46	0.29	0.24	−0.29	0.69	0.27
CBCT (n=16)	−0.09	−0.49	0.48	0.28	−0.28	−0.75	0.06	0.27	0.15	−0.34	0.73	0.27

### D. ongoing system performance

Safe and effective SABR delivery depends on accurate image guidance. Essential QA of IGRT systems includes regular assessment of the coincidence of the lasers, kV imaging, and MV delivery systems. Table 11 shows the offset between Vero lasers and the kV imaging system axis, measured monthly from installation to present. Agreement is within nationally accepted tolerance for stereotactic procedures (≤1 mm),^(12)^ though a small systematic effect (0.2−0.3 mm) can be observed in the lateral and vertical directions. Because localization lasers are mounted on the Vero gantry and rotate with the gantry and ring, the procedure for realigning the lasers is rather labor intensive. We have adopted an institutional tolerance of 0.5 mm for realignment of the lasers. Similarly, Table 12 shows the offset between kV imaging and MV delivery axes, measured monthly from installation to present. In this case, ΔX and ΔY refer to the in‐plane deviation of the radio‐opaque ball from the center of the $2\times 2\;{\text{cm}}^{2}$ MV field; that is, the orientation of X and Y with respect to the treatment coordinate system changes with the ring and gantry angle. Again, observed average displacements are all on the order of 0.1−0.2 mm, with standard deviations of the same magnitude. Interestingly, there is small dependence of X offset with gantry angle; no such dependence is observed with respect to either ring angle or in the Y direction. The largest deviation observed in any direction is 0.4 mm. The machine itself has an interlock that prevents operation if ΔX or ΔY exceeds 0.5 mm. To date the kV/MV coincidence has remained stable, while the lasers have been adjusted once. Vero dosimetric performance measured daily since the time of installation is summarized in Table 13. All parameters are well within acceptable levels and have remained remarkably stable; no trends have been observed.

**Table 11:** Average and standard deviation (STD) offset between Vero lasers and central axis of the kV imaging system, evaluated monthly since installation (n=22), in the three principal directions

		*Offset (mm)*		
*Direction*	*Ave*	*Min*	*Max*	*STD*
Lateral	−0.210	−0.7	0.4	0.279
Longitudinal	−0.029	−0.7	0.4	0.257
Vertical	0.314	0.0	0.7	0.213

**Table 12:** Average and standard deviation (STD) offset between central axes of kV and MV imaging systems, evaluated monthly since installation (n=22), reported as a function of gantry and ring angle

		*Offset (mm)*			
*Gantry Angle*	*Ring Angle*	*ΔX*	*STD*	*ΔY*	*STD*
270	0	0.220	0.089	0.070	0.134
0	0	0.130	0.134	0.120	0.121
90	0	−0.070	0.134	0.120	0.152
180	0	0.000	0.112	0.110	0.121
180	20	−0.040	0.105	0.110	0.279
90	20	−0.089	0.138	0.200	0.117
0	20	0.150	0.110	0.100	0.103
270	20	0.220	0.089	0.090	0.101
270	340	0.220	0.111	0.130	0.101
0	340	0.070	0.149	0.140	0.137
90	340	−0.090	0.102	0.170	0.147
180	340	−0.020	0.089	0.100	0.101

**Table 13:** Vero dosimetric parameters measured daily (n=426) since the time of installation

*Dosimetric Parameter*	*Average*	*STD*	*Min*	*Max*
Output Deviation %	Baseline	0.40	−1.52	2.78
Flatness %	0.14	0.12	−0.25	0.61
Axial Symmetry %	−0.17	0.35	−1.55	1.18
Transverse Symmetry %	0.08	0.26	−0.88	0.95

### C. Clinical applications

**Figure 8:**
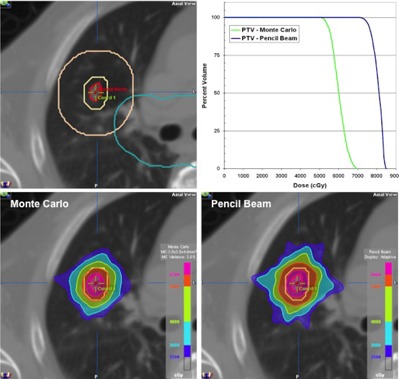
SBRT plan for a patient with small lung metastases, showing ITV, PTV, PTV+2 cm, and central zone contours (top left). Dose calculation performed using Monte Carlo (bottom left) and pencil beam (bottom right) algorithms, with the corresponding DVH for each (top right).

Figure 8 shows a treatment plan for a patient with a small (8−10 mm) peripheral lung metastasis. A dose of 54 Gy delivered in 3 fractions was prescribed such that at least 95% of the PTV received 54 Gy, and 100% of the PTV at least 51.3 Gy. Ten conformal beams were used, including six with a nonzero ring angle (i.e., noncoplanar); this produces a dose distribution that is compact and relatively isotropic in all directions. Based on previous commissioning results, the Monte Carlo algorithm was used for dose calculation. DVH for MC and PB calculations are shown for comparison. The PB algorithm predicts a dose more than 25% greater than that calculated using MC; that is, use of the PB algorithm in this case would result in a severe underdose.

**Figure 9:**
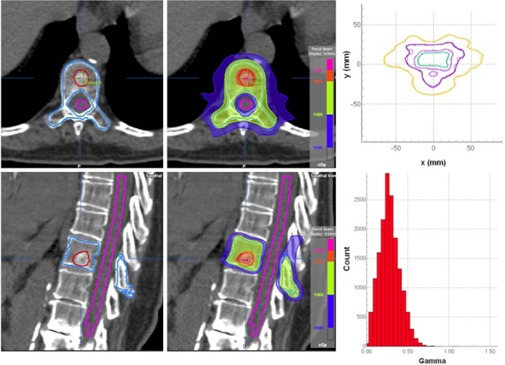
SBRT plan for a patient with spinal metastases, showing GTV, CTV and cord contours (left) and dose distribution showing 20, 14, and 10 Gy levels (middle). The calculated dose distribution superimposed on film dosimetry is shown at top right, with the resulting gamma distribution (3%/3 mm) at bottom right.

Figure 9 shows a treatment plan for a patient with metastasis to the thoracic spine. Treatment was delivered in a single fraction, using 13 coplanar IMRT beams distributed uniformly over 360°. A dose of 20 Gy was prescribed to the GTV, and 14 Gy to the CTV which encompassed the entire vertebral body, spinous process, and both transverse processes. As such, the CTV completely encircled the spinal cord. The plan shows excellent coverage at both dose levels and excellent sparing of the spinal cord, which received a maximum dose of 9.5 Gy. As part of patient specific QA, the measured dose distribution is shown superimposed on calculation with resulting 2D gamma results.

## IV. DISCUSSION

This study summarizes the commissioning process and initial clinical experience of a new treatment device developed for delivery of image‐guided stereotactic ablative radiotherapy (SABR). Despite a completely new linac and new treatment head design, Vero beam data characteristics are remarkably similar to those of other linacs operating at 6 MV.^38^, ^42^, ^43^, ^44^ Agreement between dosimetry measurement and calculation is exceptionally good, both in Solid Water and in a heterogeneous thorax phantom.

The shortcoming of PB algorithms to accurately predict dose in low density media has been described previously.^27^, ^32^, ^45^, ^46^, ^47^, ^48^, ^49^ In a recent study of 304 irradiations performed at 221 different institutions, Kry et al.^(49)^ observed an overestimate of 4.9% in PB algorithms compared to measurement in the Radiological Physics Center (RPC) thorax phantom used for RTOG credentialing; these results agree favorably with those reported here.

Recent guidance documents emphasize the importance of end‐to‐end testing that evaluate both IGRT localization and dosimetry aspects in an integrated manner.^13^, ^14^, ^50^ Several groups have evaluated positioning accuracies of independent IGRT methodologies utilizing floor mounted stereotactic X‐ray localization and gantry‐mounted volumetric (CBCT) imaging. Ma et al.^(51)^ reported residual RMS errors from 0.17 to 0.30 mm (σ from 0.17 to 0.29 mm) for stereotactic X‐ray localization and 0.35 to 0.50 mm (σ from 0.33 to 0.48 mm) for CBCT localization. Kim et al.^(38)^ reported average errors from −0.2 to −0.8 mm (σ from 0.2 to 0.4 mm) for stereotactic X‐ray localization and 0.0 to 0.6 mm (σ from 0.5 to 0.7 mm) for CBCT localization. While wider error distributions were observed for CBCT localization in both studies, differences between the two methodologies did not reach statistical significance. In this study, stereotactic X‐ray and CBCT localization accuracy was assessed through 28 independent hidden‐target tests in special head and thorax phantoms. No systematic errors were observed with either methodology, and error distributions were similar to those reported in the earlier studies.

## V. CONCLUSIONS

Vero characteristics and performance are well suited for stereotactic‐ablative radiotherapy application in which target localization and dose conformality and compactness are paramount. The ring gantry design provides unique degrees of freedom for beam delivery, which is important for creating the isotropically conformal and compact dose distributions that are needed in SABR, and exhibit high mechanical stability that contributes to excellent localization accuracy. Stereotactic X‐ray and CBCT imaging are tightly integrated with delivery systems and can facilitate sophisticated treatment techniques, such as gimbal tracking. We have demonstrated excellent agreement between iPlan calculations and measurement, through systematic benchmarking in Solid Water, specialized anthropomorphic phantoms, and the RPC credentialing phantoms. To date, Vero has been used for SABR in lung, liver, spine, and other tumor sites. Treatments require between 30 and 60 minutes, depending on the dose, treatment technique, and number of intrafraction imaging sessions. Potential shortcomings of the Vero include a relatively low dose rate, 500 MU/minute, which can extend treatment times, and 0.5 cm wide MLC leaves, which may preclude optimal field shaping for very small tumors.
